# Retraction: TDP-43 causes neurotoxicity and cytoskeletal dysfunction in primary cortical neurons

**DOI:** 10.1371/journal.pone.0252752

**Published:** 2021-06-01

**Authors:** The PLOS ONE Editors

Following the publication of this article [1], concerns were raised regarding the results presented in Fig 4. Specifically,

- In Fig 4A-D, there are cells within each panel that are highly similar to one another. In many cases, the background around the similar cells appears discontinuous with neighbouring background such that there appear to be rectangles of differently shaded background around these cells. Underlying data provided by the authors confirm that additional cell images were pasted into all four figure panels, affecting the number of bright and dark cells shown. Furthermore, the underlying data provided for Fig 4C did not match the panel presented in the published figure, raising further concerns about the validity of the data reported in this panel.
- The number of TUNEL-positive cells in the underlying images provided by the authors do not appear to reflect the quantitative trend reported in the 72-h histogram (Fig 4E) for panels B, C, and D. Furthermore, the underlying data provided to support this figure do not indicate the counted TUNEL-positive cells were normalised to total cell numbers, as was reported in the Methods section.

The authors clarified that the quantification of the Fig 4 results took place under the microscope and that only representative images were saved. The authors stated that the data underlying the other results reported in this article are still available.

This issue was reviewed by the Research Integrity Office and Dean for Research Governance, Ethics and Integrity at Kings College London. The institution did not find evidence of intent to deceive but noted some issues around lack of awareness of good practice standards.

Overall, the data and comments provided did not fully resolve the above concerns nor provide adequate assurance as to the reliability of the results reported in Fig 4. In light of these issues, the PLOS ONE Editors retract this article.

PB agreed with the retraction and apologises for the issues with the published article. CS and SG either did not respond directly or could not be reached.
